# Low abundance of sharks and rays in baited remote underwater video surveys in the Arabian Gulf

**DOI:** 10.1038/s41598-018-33611-8

**Published:** 2018-10-22

**Authors:** Rima W. Jabado, Shamsa M. Al Hameli, Edwin M. Grandcourt, Shaikha S. Al Dhaheri

**Affiliations:** 1 Environment Agency - Abu Dhabi, P.O. Box 45553, Al Mamoura Building, Murour Road, Abu Dhabi, United Arab Emirates; 2 Gulf Elasmo Project, P.O. Box, 29588 Dubai, United Arab Emirates

## Abstract

Data on the diversity and relative abundance of elasmobranchs (sharks and rays) in the Arabian Gulf have been limited to fishery-dependent monitoring of landing sites. Understanding the diversity and abundance of sharks and rays is, however, crucial to inform policy and management plans. Baited Remote Underwater Video Surveys (BRUVS) were conducted in 2015–2016 across the United Arab Emirates Arabian Gulf waters encompassing a range of depths and habitat types. Data from 278 BRUVS (757 hours soak time) were analysed to gather information on diversity, relative abundance, species distribution, and habitat associations. Surveys recorded 213 individuals from 20 species of sharks and rays at 129 stations. The frequency of occurrence of species usually discarded by fishers such as the Arabian carpetshark (*Chiloscyllium arabicum*) and stingrays (*Himantura* spp.) was high, accounting for 60.5% of observed elasmobranchs. Despite the large survey area covered and extensive sampling effort, the relative abundance of sharks and rays was low at 0.28 elasmobranchs per hour, 0.13 sharks per hour, and 0.15 rays per hour. This CPUE was reduced to one of lowest recorded abundance on BRUVS from around the world when removing the two discarded species from the analysis (0.11 elasmobranchs per hour). These results likely reflect the intense fishing pressure and habitat loss contributing to population declines of many elasmobranchs in the Arabian Gulf. Findings provide a baseline for future work and can support the design of conservation strategies for sharks and rays in the UAE.

## Introduction

Populations of many species of elasmobranchs (sharks and rays) have drastically declined worldwide due to overfishing and habitat loss leading to growing concerns over their long-term sustainability^1,2^. Our understanding of population trends and ecology of these species largely relies on a combination of fishery-dependent sources (catch per unit effort (CPUE)) or extractive fishery-independent techniques (e.g., trawl, gillnet, and longline surveys) to estimate relative abundance^3^. These methods form the basis of fisheries management but can result in abundance, catchability, and size selectivity biases^4,5^. Furthermore, these approaches have a limited ability to survey high complexity habitats such as coral reefs, do not provide accurate information on non-target species of the various fisheries sampled (i.e., bycatch), and often fail to record rare and threatened species^4–6^.

Beyond the range of commercial and recreational fisheries, fundamental information pertaining to diversity, distribution, and abundance estimates of sharks and rays is crucial for the development of effective management and conservation initiatives^7,8^. However, data collection for these species is especially difficult because most are highly mobile, have ontogenetic shifts in habitat preferences, and have broad geographic ranges^9,10^. Although there are limited resources and funding to undertake such large scale sampling, the prioritisation of these data is critical to fisheries management and to evidence-based conservation planning^7^.

A growing number of studies have considered alternative methods to independently assess elasmobranch populations and reduce current data gaps^6,11^. The baited remote underwater video system (BRUVS) technique is now established as a cost-effective, non-extractive means of providing a standardized, non-invasive, non-extractive, and non-destructive fishery-independent sampling method to (1) estimate the relative abundance of elasmobranchs across geographically wide areas as well as a range of habitats and depths that might otherwise be inaccessible; (2) provide estimates of species richness; (3) analyse species-specific behaviours; and (4) determine size and biomass when using stereo-cameras^4,5,8,11–16^. This method uses bait to attract individuals into the field of view of a camera so that species can be identified and individuals counted. This approach often complements other traditional methods to enhance the scope and capability of monitoring and stock assessment programs^13^. Indeed, studies indicate that BRUVS produce similar relative abundance estimates and species composition to other fishery-independent techniques such as longline surveys^5^, even though a greater amount of replicates and amount of time might be required^12^.

The Arabian Sea and its adjacent waters has recently been highlighted as one of the regions with the most threatened shark and ray populations in the world^17^. Much of the data available from the region, and particularly in the Arabian Gulf, stem from fishery-dependent trawl research surveys or monitoring of landings at various sites (e.g.,^18,19^). Here, we report on the results from the first BRUVS to be deployed in the Arabian Gulf waters of the United Arab Emirates (UAE) with the aim to (1) assess the relative abundance of sharks and rays across UAE Gulf waters using fishery-independent methods; (2) collect data on shark and ray distribution patterns and habitat associations; and (3) investigate how factors such as season, depth, habitat, and geographic stratum can influence the presence of sharks and rays.

## Results

**Figure 1:**
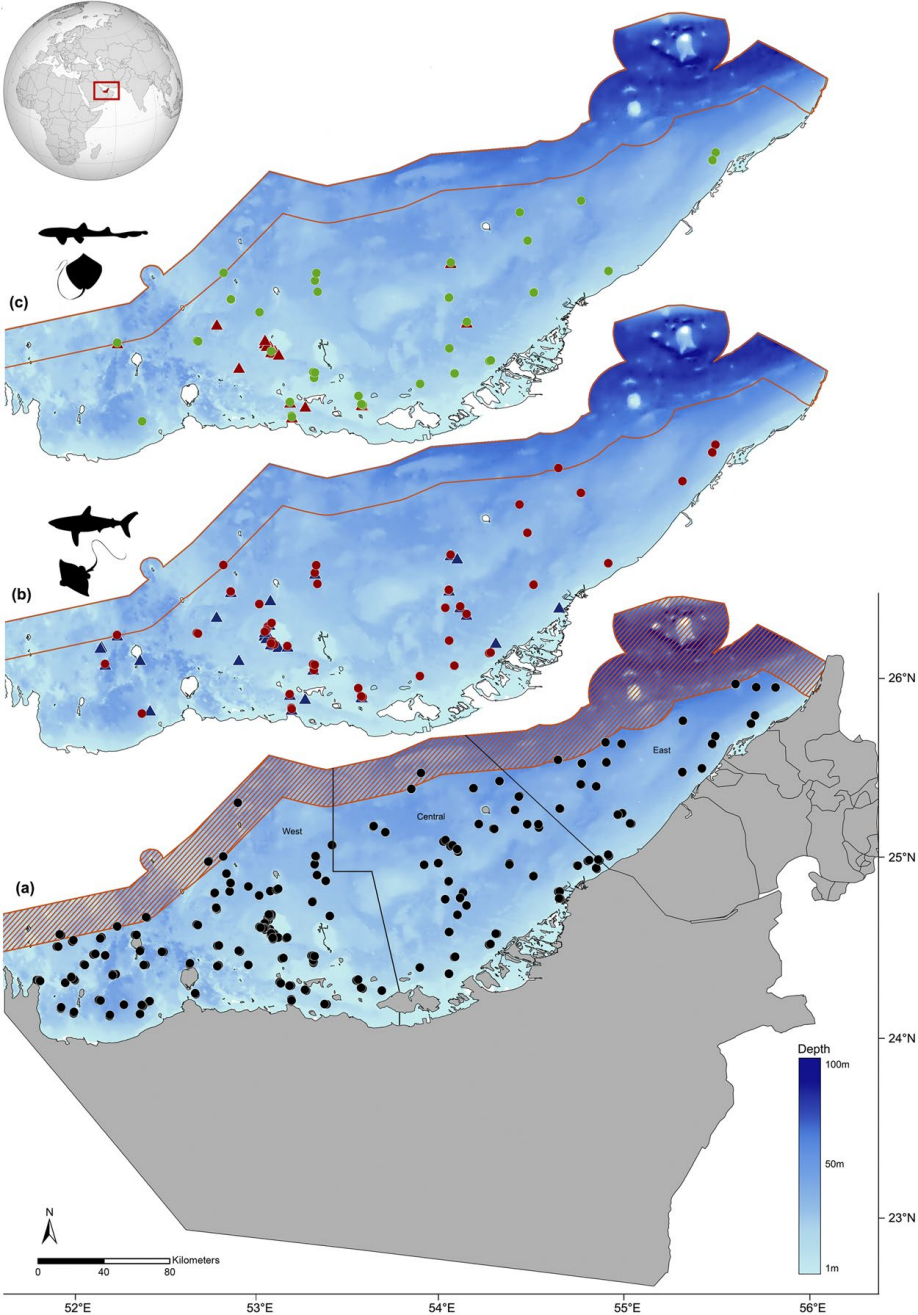
Map of the United Arab Emirates showing locations of (**a**) stations (n = 278) where baited remote underwater video surveys were deployed = ●, (**b**) location of shark =  and ray =  sightings, and (**c**) location of Arabian carpetshark *Chiloscyllium arabicum* = and stingray *Himantura* spp. = sightings.

**Figure 2:**
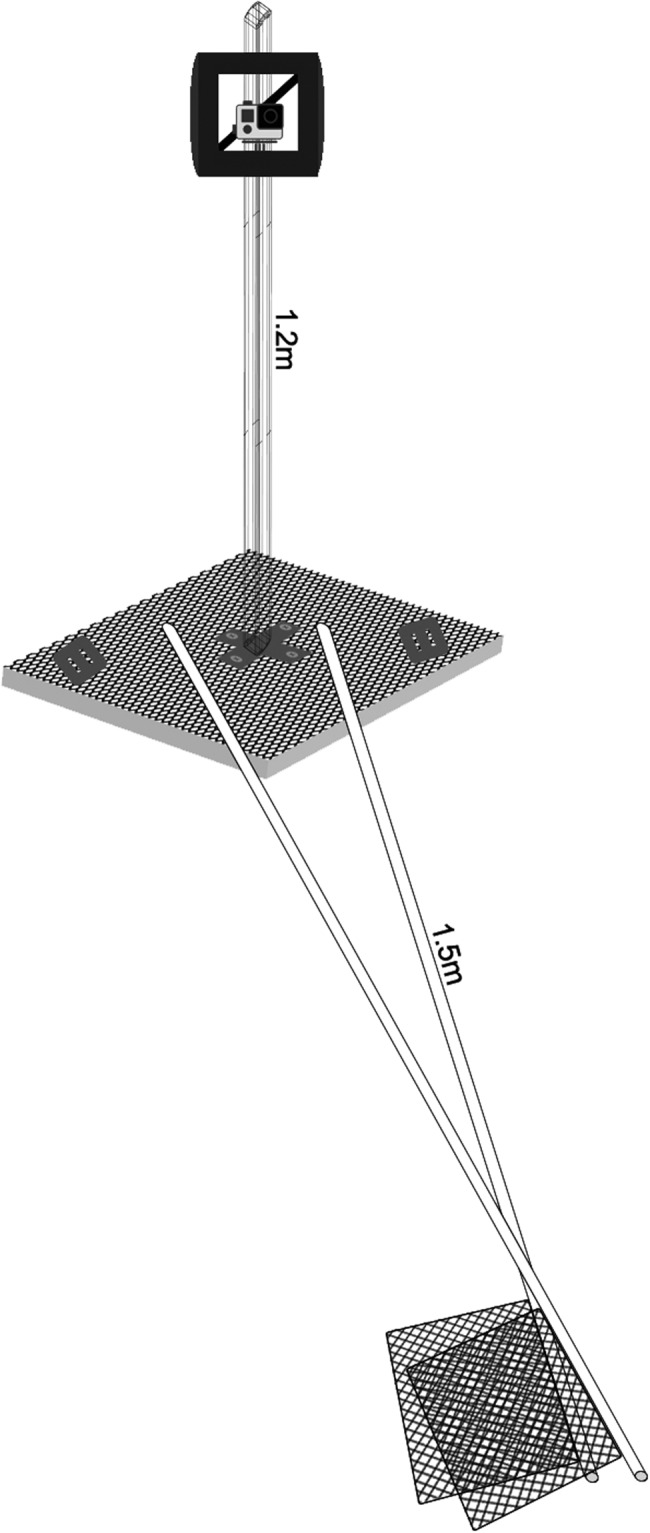
Baited Remote Underwater Video Survey unit with GoPro Hero 4 mounted, bait arm, and bait bag.

Fisheries Resource Assessment Surveys (FRAS) (see *Methods* for details) were undertaken in May-June 2015, April to June 2016, and October to December 2016. A total of 283 BRUVS were deployed during this period. Of these, 278 sites were used in the analysis (Fig. 1a); four were discarded due to inadequate visibility (<1.5 m) and one BRUVS unit was stolen (Fig. 2). The total number of video hours was 757.2 hours with a minimum of 64 minutes, a maximum of 3.2 hours, and a mean deployment time of 2.1 hours (±0.44 hours SD). All deployments were spread throughout daylight hours from 06.55–18.15 hrs. Deployment site depths ranged from 1 to 41 m, with a mean sampling depth of 16.6 m (±8.5 m SD). Details of deployments based on the various factors used in the analysis are provided in Table 1.

**Table 1:** Summary of the number of Baited Remote Underwater Surveys (*n* BRUVS) completed in 2015 and 2016 by *Depth*, *Habitat* and *Strata*.

Survey period	n BRUVS	Depth		Habitat			Strata		
		≤10 m	>10 m	Mud	Sand	Other	West	Central	East
Spring 2015	26	26	0	0	2	24	26	0	0
Spring/Summer 2016	134	49	85	58	38	38	107	23	4
Fall/Winter 2016	118	50	68	51	51	16	30	53	35
**Total**	**278**	**125**	**153**	**109**	**91**	**78**	**163**	**76**	**39**

### Species richness and diversity

**Figure 3:**
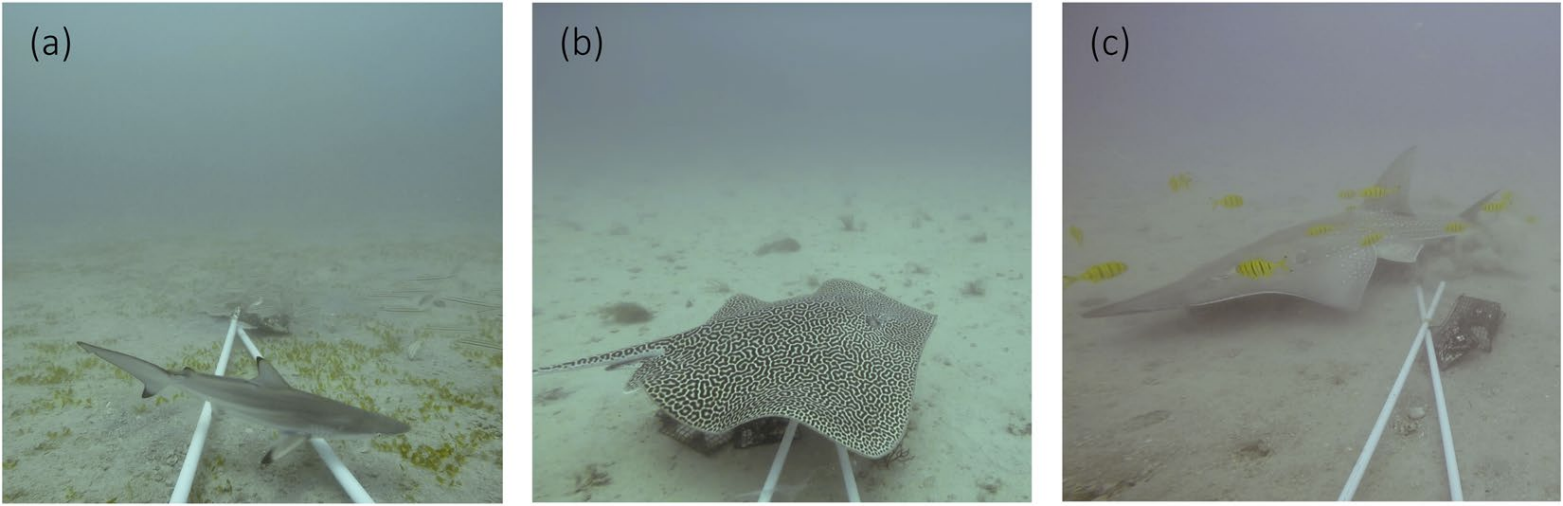
Images of elasmobranchs captured by the baited remote underwater video systems in the Arabian Gulf, (**a**) spottail shark *Carcharhinus sorrah*, (**b**) leopard whipray *Himantura leoparda*, (**c**) smoothnose wedgefish *Rhynchobatus laevis*.

**Figure 4:**
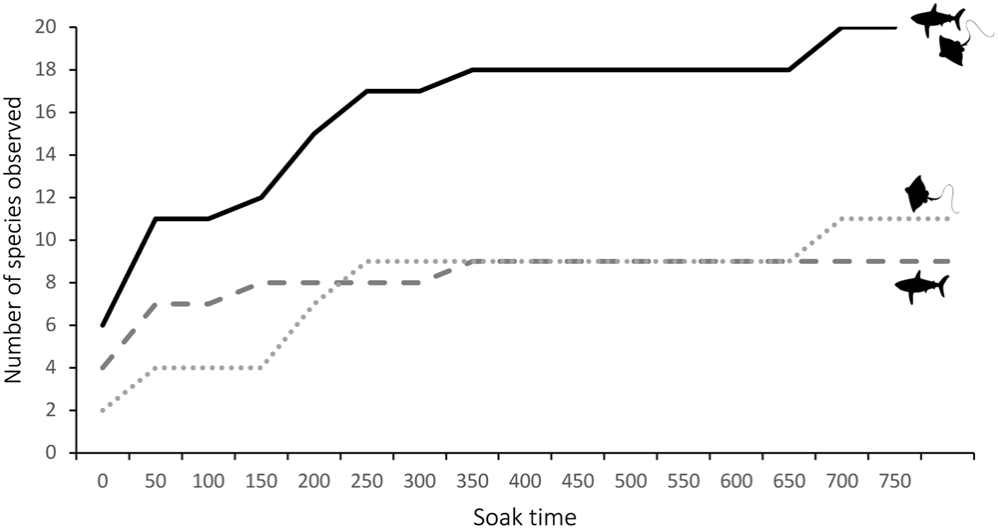
Cumulative number of elasmobranchs (➖), sharks (), and rays () recorded for each 50-hour soak time interval on the baited remote underwater video systems in the Arabian Gulf.

Two-hundred and thirteen individual sharks and rays were observed in which 99 sharks and 114 rays were recorded (Fig. 1, Table 2). From the 278 deployments, sharks and rays were observed on 129 (46.4%) of the BRUVS with 64 deployments (23%) recording sharks, and 88 (31.6%) recording rays. Of the identified individuals (82.4% of individuals observed), a total of 20 shark and ray species, belonging to eight families from 15 genera were recorded (Fig. 3). These included nine species of sharks from two families and five genera and 11 species of rays from six families and 10 genera (Table 2). This represents 29.4% of elasmobranch species known to occur in Arabian Gulf waters (32 shark species of which 28.1% were observed and 36 ray species of which 30.5% were observed). The cumulative curve indicates that after approximately 300 hours of soak time, we had adequately sampled species richness and few additional species were recorded in the remaining survey time (Fig. 4).

**Table 2:** Summary of results of Baited Remote Underwater Video Surveys (BRUVS) with a summary of shark and ray species observed during deployments, abundance (sum (Σ) of MaxN and % MaxN), depth ranges (m), habitat associations (S – Sand, M – Mud, O – Other; and MaxN by type), seasons (SS – Spring/Summer, FW – Fall/Winter; and MaxN for each), and estimated size ranges (cm) as total length (TL) or Disc Width (DW).

	Common name	Species name	Σ MaxN	% Occurrence	Depth	Habitats (MaxN)	Seasons (MaxN)	Size range
**SHARKS**								
Carcharhinidae	Pigeye shark	*Carcharhinus amboinensis*	1	0.5	11	M (1)	FW (1)	>150 TL
	Whitecheek shark	*Carcharhinus dussumieri*	17	8	11–24	M (3), S (2), O (1)	SS (1)– FW (3)	<100 TL
	Blacktip shark	*Carcharhinus limbatus*	6	2.8	9–24	M (2), O (1)	SS (1) – FW (2)	<100 TL
	Blacktip reef shark	*Carcharhinus melanopterus*	2	0.9	1–6	O (1)	SS (1)	<100 TL
	Spottail shark	*Carcharhinus sorrah*	16	7.5	3–30	M (2), S (2), O (1)	SS (2) – FW (2)	<100 TL
	Sliteye shark	*Loxodon macrorhinus*	3	1.4	13–20	M (1), S (1), O (1)	SS (1)- FW (1)	<100 TL
	Sharptooth lemon shark	*Negaprion acutidens*	1	0.5	6	O (1)	SS (1)	>100 TL
	Milk Shark	*Rhizoprionodon acutus*	4	1.9	13–20	M (1)	SS (1)- FW (1)	<100 TL
	Whaler shark	*Carcharhinus* sp.	2	0.9	20–22	M (1), O (1)	FW (1)	<100 TL
Hemiscyllidae	Arabian carpetshark	*Chiloscyllium arabicum*	49	23	3–23	M (2), S (1), O (3)	SS (3) – FW (2)	<100 TL
***Total sharks***			**99**	**47**.**3**				
**RAYS**								
Aetobatidae	Spotted eagle ray	*Aetobatus ocellatus*	6	2.8	4–20	S (1), O (1)	SS (1)	n/a
Dasyatidae	Leopard whipray	*Himantura leoparda*	7	3.3	11–22	M (1), S (1), O (1)	SS (1) – FW (1)	80–100 DW
	Reticulate whipray	*Himantura uarnak*	67	31.5	5–37	M (2), S (3), O (2)	SS (2) – FW (3)	40–>100 DW
	Pink whipray	*Pateobatis fai*	2	0.9	17–29	M (1)	FW (1)	40–100
	Cowtail ray	*Pastinachus* sp.	6	2.8	5–13	M (1), S (1), O (1)	SS (1) – FW (1)	40–100
	Blotched fantail ray	*Taeniurops meyeni*	1	0.5	19	S (1)	SS (1)	60–80 DW
Glaucostegidae	Halavi guitarfish	*Glaucostegus halavi*	1	0.5	22	M (1)	SS (1)	>100 TL
Gymnuridae	Longtail butterfly ray	*Gymnura poecilura*	1	0.5	17	S (1)	SS (1)	30–40 DW
Rhinidae	Bowmouth guitarfish	*Rhina ancylostoma*	1	0.5	14	0 (1)	SS (1)	>100 TL
	Smoothnose wedgefish	*Rhynchobatus laevis*	2	0.9	17–25	S (1)	SS (1)	>200 TL
	Wedgefish	*Rhynchobatus* sp.	1	0.5	21	M (1)	FW (1)	100–200 TL
Rhinopteridae	Cownose ray	*Rhinoptera* sp.	3	1.4	5–14	O (2)	SS (2)	40–100 DW
*Unidentified*		*Himantura* sp.	14	6.6	5–37	M (1), S (1), O (2)	SS (2) – FW (1)	n/a
***Total rays***			**114**	**52**.**7**				
**TOTALS**			**213**	**100**				

n/a indicates individuals did not approach the bait arm to allow for size estimates.

The frequency of occurrence of most species was low except for *Chiloscyllium arabicum* and *Himantura* spp. that dominated during the surveys. These were the most frequently observed species accounting for 60.5% of all elasmobranchs, 49.5% of identified sharks, and 70.1% of identified rays, respectively. Additional dominant shark species observed included the whitecheek shark (*Carcharhinus dussumieri*) (17.2% of identified individuals and the highest MaxN recorded of 3 sharks in one video frame) and the spottail shark (*C*. *sorrah*) (16.2% of identified individuals). For rays, *H*. *leoparda* and *Pastinachus* spp. accounted for 6.1% and 5.3% of identified rays, respectively. Most species were only recorded once throughout the surveys including the pigeye shark (*C*. *amboinensis*), sharptooth lemon shark (*Negaprion acutidens*), Halavi guitarfish (*Glaucostegus halavi*), and the blotched fantail ray (*Taeniurops meyeni*). A total of 16 sharks and rays recorded could not be identified due to their distance from the BRUVS unit.

It was not possible to accurately estimate lengths of individual sharks and rays, especially if these were observed in the distance. However, at proximity, the majority of sharks (97.9%) were notably smaller than the bait arm (1.5 m). The majority of *Himantura* spp. for which we were able to record an estimate of size (n = 68) were between 50 cm and 100 cm DW (76.4%) with 8.8% larger than 100 cm DW, 76.4% between 50 cm and 100 cm DW, and 5.8% smaller than 50 cm DW. The largest shark recorded was *C*. *amboinensis* at over 150 cm TL while the largest ray was a smoothnose wedgefish (*Rhynchobatus laevis*) estimated at over 200 cm TL. The smallest rays were estimated at around 50 cm TL. Sex could only be determined for 56 *Himantura* spp. of which 60.7% were male and 39.3% were female.

Analysis of tapes showed there was no apparent relationship between soak time and the probability of a shark or ray appearing in the video. Individuals were sighted from the second minute (2.6 mins) of deployment and until 160.5 mins had elapsed with a mean number of 59.1 (±41.4 SD) minutes elapsed to first sighting.

### CPUE

**Figure 5:**
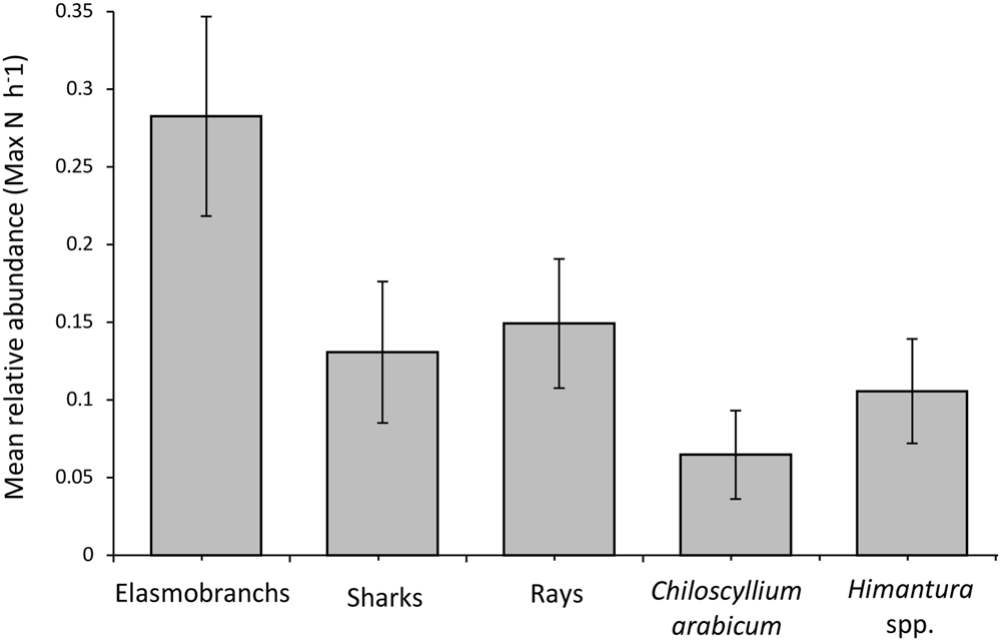
Mean relative abundance (MaxN h^−1^ ± SE) of Elasmobranchs, Sharks, Rays, Arabian carpetshark *Chiloscyllium arabicum*, and stingrays *Himantura* spp. determined by baited underwater video surveys.

**Figure 6:**
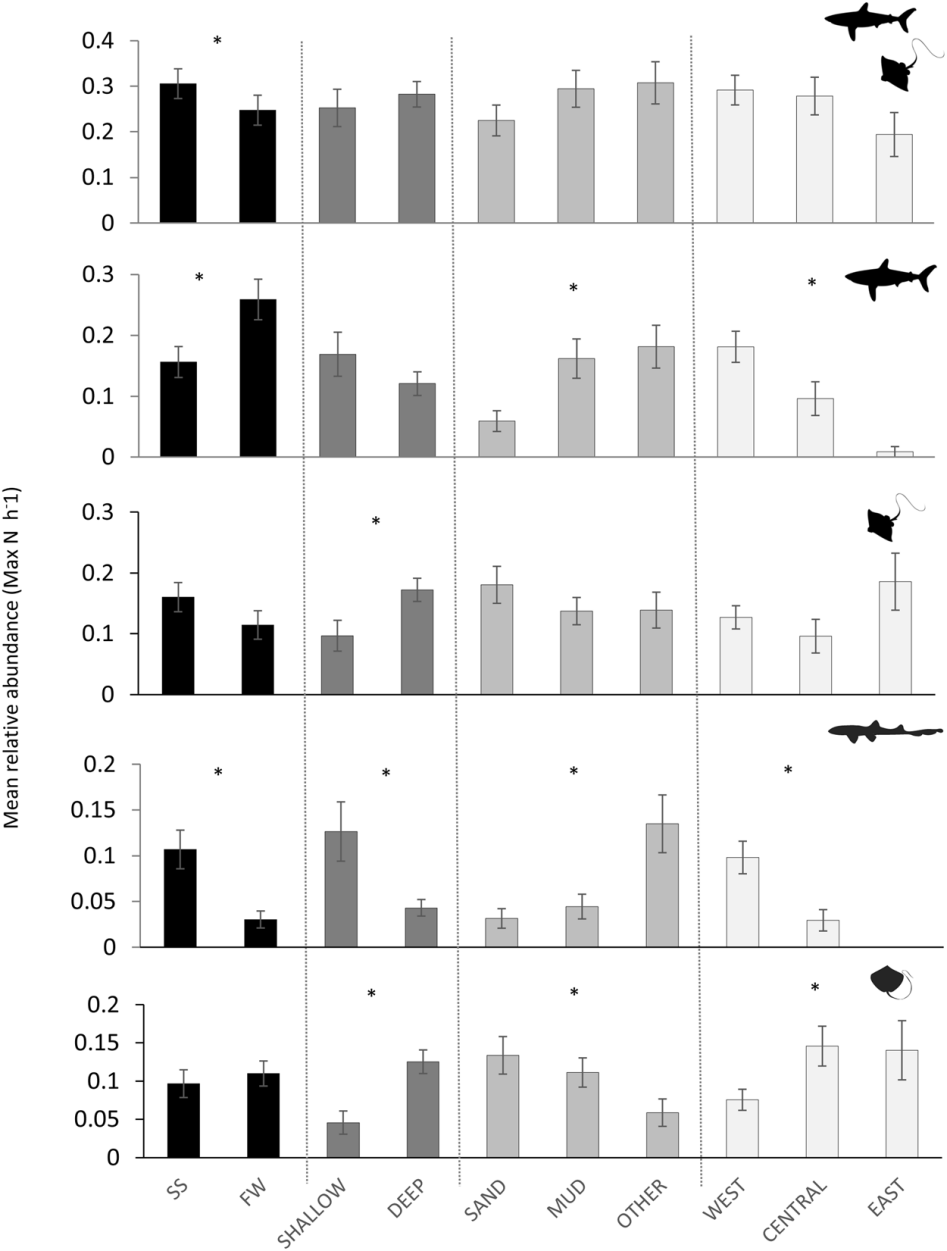
Mean relative abundance (MaxN h^−1^ ± SE) of Elasmobranchs, Sharks, Rays, Arabian carpetshark *Chiloscyllium arabicum*, and stingrays *Himantura* spp. determined by baited underwater video surveys in relation to *Season* (SS: Spring/Summer, FW: Fall/Winter), *Depth* (Shallow: ≤10 m, Deep: >10 m), *Habitat* (Sand, Mud, Other), and *Strata* (West, Central, East). * Indicates a significant difference (p < 0.05) between groups.

Overall, the CPUE obtained by BRUVS surveys was relatively low (0.28 elasmobranchs per hour, 0.13 sharks per hour, and 0.15 rays per hour) (Fig. 5). This CPUE was significantly reduced when excluding observations of *C*. *arabicum* and *Himantura* spp. from the analysis (0.11 elasmobranchs per hour and 0.06 sharks per hour and 0.04 rays per hour respectively). Differences in the relative abundance of elasmobranchs, sharks, rays, *C*. *arabicum* and *Himantura* spp. based on *Season*, *Depth*, *Habitat*, and *Strata* are presented in Fig. 6(a–e) and Table 3.

**Table 3:** Results of ANOVA testing the effect of *Season* (SS: Spring/Summer, FW: Fall/Winter), *Depth* (Shallow: ≤10 m, Deep: >10 m), *Habitat* (S: Sand, M: Mud, O: Other), and *Strata* (W: West, C: Central, E: East) on the relative abundance of Elasmobranchs, Sharks, Rays, the Arabian carpetshark (*Chiloscyllium arabicum*) and stingrays (*Himantura* spp.); df = degrees of freedom; bold *p* values denotes significance at p < 0.05.

		df	X^2^	*p*	df	X^2^	*p*	df	X^2^	p	df	X^2^	*p*	df	X^2^	*p*
**Season**	(SS × FW)	1	4.322	**0**.**038**	1	4.421	**0**.**036**	1	0.242	0.623	1	0.702	**0**.**000**	1	12.513	0.402
**Depth**	(S × D)	1	0.355	0.551	1	1.772	0.183	1	6.526	**0**.**011**	1	7.194	**0**.**007**	1	9.047	**0**.**003**
**Habitat**	(S × M × O)	2	0.755	0.686	2	8.968	**0**.**011**	2	2.250	0.325	2	12.809	**0**.**002**	2	6.947	**0**.**031**
**Strata**	(W × C × E)	2	1.109	0.574	2	15.327	**0**.**000**	2	5.556	0.062	2	13.940	**0**.**001**	2	9.161	**0**.**010**
***Pairwise tests***			**U**	***p***		**U**	***p***		**U**	***p***		**U**	***p***		**U**	***p***
**Habitat**	(S × M)		4685	0.533		4337	**0**.**038**		4548	0.280		4831	0.689		4743	0.594
	(S × O)		3306	0.401		2848	**0**.**002**		3175	0.157		2927	**0**.**003**		2954	**0**.**010**
	(M × O)		4122	0.788		3918	0.305		4075	0.639		3526	**0**.**004**		3646	**0**.**030**
**Strata**	(W × C)		5987	0.651		5417	**0**.**044**		5336	**0**.**035**		5431	**0**.**018**		5155	**0**.**005**
	(W × E)		2940	0.422		2274	**0**.**000**		2753	0.104		2515	**0**.**002**		2724	**0**.**049**
	(C × E)		1318	0.289		1261	**0**.**022**		1472	0.946		1365	0.073		1455	0.849

**Figure 7:**
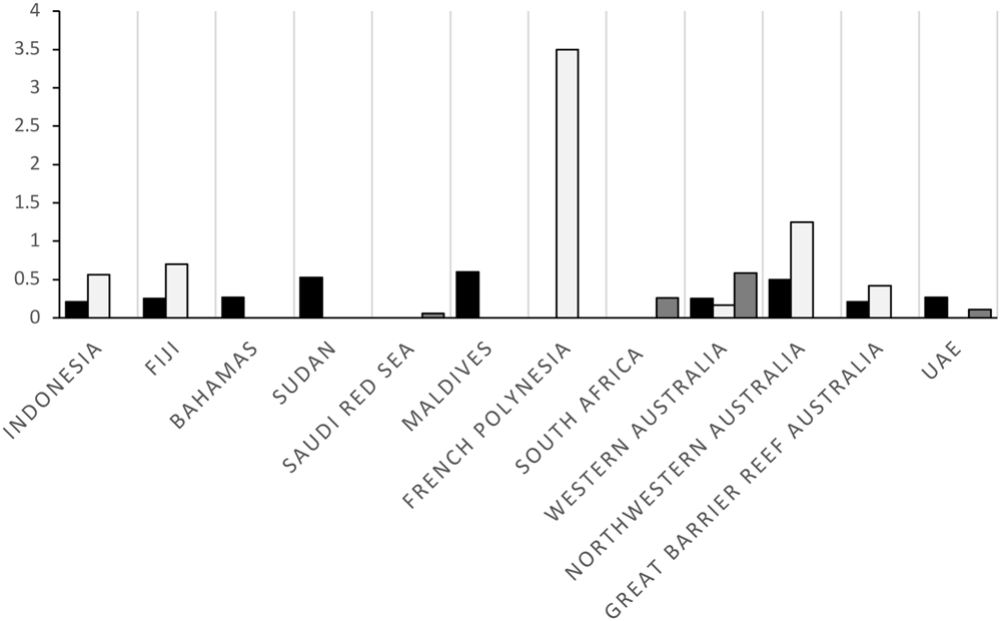
Comparison of mean relative abundance (MaxN h^−1^) published from BRUVS studies from around the world for sharks in fished areas (black), sharks in no-take zones (light grey), and elasmobranchs in fished areas (dark grey). For the UAE, the dark grey column represents the CPUE of sharks and rays after removing the Arabian carpetshark (*Chiloscyllium arabicum*) and stingrays (*Himantura* spp.) from the analysis.

This CPUE recorded for the southern Arabian Gulf was between one to two orders of magnitude lower than published estimates obtained from reef systems in French Polynesia, Australia, Fiji, the Bahamas, and Indonesia (Fig. 7).

## Discussion

This survey is the first to estimate the relative abundance of sharks and rays using BRUVS in the Arabian Gulf and across the whole of UAE Gulf waters. It allowed a cost-effective and rapid means to sample across a broad spatial scale and range of habitats. Results clearly indicate a low relative abundance of elasmobranchs across the southern Arabian Gulf with CPUE levels up to two orders of magnitude lower than those recorded using comparable BRUVS techniques in other regions of the world (e.g., Maldives^20^, French Polynesia^6^, Western Australia^5^, and Sudan^21^). When removing *C*. *arabicum* and *Himantura* spp. from our analysis, species known to be discarded by fishers in the UAE^18,22^, our CPUE is reduced by more than half and ranges closer to the alarmingly low CPUE results from the northern Saudi Arabian Red Sea^21^. While there are few historical baselines of elasmobranch abundance from the Arabian Gulf, several studies have already highlighted the increasing fishing pressure on these species in the region with population declines as well as changes in the species composition and sizes of individuals landed (e.g.^18,19^). Overfishing is the most plausible explanation for the almost complete absence of large sharks during these surveys and the overall low diversity of species. These findings are consistent with the hypothesis that sharks and rays in the Arabian Gulf and broader Arabian Sea and adjacent waters region are amongst the most threatened in the world^17^. Despite the UAE having developed legislation to regulate shark fishing (including species-specific protections), most species (especially sharks) are still landed as bycatch due to an overlap between the seasonal shark fishing ban and open gillnet fishing season^22^. Unsustainable fishing and overexploitation of elasmobranch resources, coupled with weak enforcement of fishing policies, are widespread in the region and these low CPUE numbers are likely a reflection of stocks that have been depleted from over two decades of overfishing^21,23^.

Only 29.4% of elasmobranch species known from fisheries-dependent surveys to occur in Arabian Gulf waters were recorded using BRUVS^17,24,25^. Other surveys along the Great Barrier Reef in Australia, Indonesia, and South Africa also recorded less than 50% of known elasmobranch diversity^15,26,27^. This low diversity in BRUVS could be due to several factors including the behavior of animals in the presence of bait, or environmental variables such as depth gradients or habitats surveyed^15,26^. Firstly, our BRUVS recorded during the day and some elasmobranch species exhibit diel changes in behavior and activity^28,29^. For example, many shark species are less active during the day than at night when fishers set various fishing gear (e.g., gillnets or longlines) and catch large quantities of sharks^22,29,30^. Furthermore, fishers tend to have much longer soak times (often over 12 hours) and cover large areas and therefore are more likely to capture sharks and rays during foraging trips^27^. Also, BRUVS might be biased towards species attracted to the bait^15^, while net fishing captures individuals that are not bait-dependent^27^. We do however recognize that of the 16 unidentified animals recorded on our BRUVS, 14 were rays, highlighting that the species richness of rays could be higher than what is reported here.

Vessel and logistical constraints during FRAS prevented us from sampling inshore shallow lagoons and mangrove forests, thus potentially underestimating the abundance of species occurring in these areas. Several ray species have been observed in shallow water lagoons within UAE waters^31^ while some shark species utilize mangroves as nursery areas in other regions of the world (e.g.,^32^). Nevertheless, we sampled across the depths gradients of UAE waters (1 to 41 m), in many shallow areas, as well as structurally complex habitats (seagrass beds, soft-sediment inter-reef habitats, macro-algae), areas that also likely provide ideal habitats for many elasmobranch species. We found no significant differences in the abundance of elasmobranchs for each of these variables. It is unlikely that depth influenced the abundance of sharks, a result that is consistent with those from^16^ and^33^ who suggest that the lack of difference in abundance with depth could be due to their attraction to the bait along with their capability to disperse between different depths. Furthermore, several studies have shown that sharks are often more common at offshore sites than in inshore coastal habitats (e.g.,^15^). In the UAE, fishers have confirmed they travel to deeper waters to catch sharks as they have practically disappeared from inshore waters and it is unlikely that the limited sampling in inshore habitats would have significantly changed our CPUE results for sharks^22^. Less information is available on habitat preferences of ray species occurring in UAE waters. Further research in these shallow inshore habitats is warranted to determine which are critical sites for elasmobranchs including breeding aggregation, mating, and nursery areas.

While gear selectivity or environmental variables could also be a reason for not sampling the overall diversity of species in UAE waters, we argue that the core reason many species were not present in our surveys is due of their current low abundance in these waters. Indeed, while the relative abundance of shark species varied from that recorded during fishery-dependent surveys in the UAE, except for *C*. *arabicum*, species diversity was consistent with dominant species landed by fishers across the country. Interviews with fishers, as well as landing site surveys confirm that carpet sharks are not targeted by fishing activity in the UAE and are usually discarded^18,22^. In the UAE, over 90% of landings consist of the spottail (*C*. *sorrah*), milk (*Rhizoprionodon acutus*), common blacktip shark (*C*. *limbatus*), whitecheek shark (*C*. *dussumieri*) and slit-eye shark (*Loxodon macrorhinus*) sharks, all of which were observed in this study and therefore confirming their prevalence in these waters^18^. Indeed, because BRUVS use baits that attract sharks, they probably sample those species likely to be most affected by fishing activity^16^. On the other hand, the diversity and abundance of ray species recorded here were different than those recorded during landing site surveys. Landings data indicate that the most abundant taxa are the cownose rays (*Rhinoptera* spp.) (59.4% of ray landings), eagle rays (*Aetomylaeus* spp.) (20.6%), and wedgefishes, *Rhynchobatus* spp. (10.5%)^34^ (Jabado, unpubl. data). While several species of rays were recorded, *Himantura* spp. clearly dominated in our survey. This different species composition could be due to the fact that fishers generally discard ray catches in the UAE unless they are netted in large numbers^22^. This indicates that BRUVS have the potential to provide a better indication of diversity and abundance than fishery-dependent surveys for unwanted and discarded catch.

An understanding of habitat associations at the species level and over large spatial gradients can be a valuable approach to detect important areas for elasmobranch conservation, as well as reveal complex ecological processes such as connectivity within and across ecosystems^15^. Due to the low abundance of most species observed, it was difficult to identify spatial, seasonal, or habitat association patterns and this information was only discerned for *C*. *arabicum* and *Himantura* spp. *Chiloscyllium arabicum* sharks exhibited a strong association with shallow waters of the western region where complex habitats such as coral assemblages and seagrass beds are most prevalent. On the other hand, *Himantura* spp. were more common in the deeper waters of the eastern zone characterized by areas of sand and mud with little structurally complex habitats. These results are likely due to the habitat preferences of these two species groups which is often due to the availability of feeding and refuge sites for prey species^25,35^. Further studies are needed in the Arabian Gulf to elucidate habitat associations and use for the majority of species occurring in these waters. These data can then support assessments of the risk of species to fishing and/or habitat degradation.

The species-level identification of individuals and the determination of sizes and sex using BRUVS are challenging and common limitations^5,12,15,27,36^. On some occasions animals did not approach the camera and we were therefore only able to identify 82.4% of observed individuals. This was particularly challenging with some species of rays that need closer morphological examination to separate between similar species. Furthermore, while claspers of mature males are usually visible, discerning the sex of females or sub-adult males was challenging since it depends on the behavior of the animals in front of the camera^12^. The large majority of the animals observed here were small-bodied species with maximum total lengths or disc widths of less than 100 cm (e.g., *C*. *arabicum*, *R*. *acutus*, *C*. *dussumieri*, and longtailed butterfly ray *G*. *poecilura*)^24,25,35^ and it was therefore not possible to determine maturity levels. For some of the larger-bodied animals (e.g., *C*. *sorrah*, *C*. *limbatus*, *H*. *uarnak*, *R*. *laevis*), even though the depth of view of horizontal look-outward systems does not provide a good reference for measurements, the length of the bait arm provided a point of perspective as a scale bar allowing for coarse estimations of sizes^13^. In future studies, we recommend the use of stereo-video techniques to examine relative abundance at an intra-species resolution as they can provide accurate length and biomass estimates^12,37,38^. Indeed, the use of length-frequency estimates for selected commercially important species can provide an additional useful metric for long-term monitoring.

It is important to note that there are a number of biases associated with comparing BRUVS studies from around the world including the sampling design and deployment of units (i.e., time of day, depths), the type and quantity of bait used, and soak times^6,21^. These can highly influence estimates of relative abundance and species richness. While this BRUVS study was not effectively designed to investigate the occurrence and distribution of sharks and rays, deployment times, type and amount of bait, and survey time frames generally exceeded those reported for elasmobranchs from around the world^5,12,16,21,27,30^. One study^39^ shows that sampling precision is improved with increasing soak times as it likely enhances the probability of the bait plume intersecting individuals as they move around and attracts them to the BRUVS unit. Overall, an optimal 60-minute deployment time is proven to provide an accurate representation of species assemblages and is adequate to record 95% of species^14,37^, although sightings can continue to increase until after 180 mins of deployment^21^. It has been suggested that it takes longer for sharks to appear in the video when their abundance is low^4^. In our study, soak time varied between a minimum of one hour and up to over 3 hours, and our cumulative species curve shows that few new species were recorded after 300 hours of soak time, indicating that we adequately sampled abundance and species diversity in southern Arabian Gulf waters. We do acknowledge that water quality and visibility is likely the greatest limitation to BRUVS^13,15^. While lack of visibility due to lighting at depth is often not an issue in the shallow waters of the Arabian Gulf, the vast majority of this body of water is characterized by muddy substrates^40^. This affected visibility at certain sites when large quantities of teleost fish aggregated and created a ‘mud’ plume. Several stations had to be removed from this study due to the inability to see the bait bag (1.5 m from the camera). Some results show that there is a marginal, non-significant effect of underwater visibility on the performance of BRUVS with performance dropping only at the lowest visibility (about 1 m)^41^. Therefore, even though visibility might have been variable, there were no significant differences in CPUE between sand and mud habitats and visibility is unlikely to have affected our overall CPUE. Overcoming this limitation in Arabian Gulf waters might require the development of lift-bag systems or sampling using cameras from above.

In conclusion, this study has provided valuable data to complement existing fisheries-dependent information available in the UAE. It confirms that the BRUVS sampling technique is useful to examine the diversity, relative abundance, and habitat associations of elasmobranchs across broad spatial scales, especially in areas where the use of conventional fisheries-independent sampling tools would be difficult to implement. There is scope for the future use of this data as a baseline to monitor changes in the relative abundance of species while allowing the examination of changes in species diversity and composition over time in response to natural and anthropogenic drivers. However, combining different techniques for monitoring, including fishery-dependent and independent methods, will prove more appropriate to fully define species richness and assemblages in waters where fishing pressure is intense, fisheries discards are prevalent (e.g., carpet sharks and rays), or where rare species are known to occur. Finally, as highlighted from results in Australia^29^, if management strategies were to be developed and implemented to reverse stock declines in the Arabian Gulf, BRUVS can allow measurements of species-specific recovery patterns and demonstrate the efficacy of management strategies.

## Methods

### Study area

The Arabian Gulf is a sub-tropical and semi-enclosed basin characterized by a wide range of habitats including coral reefs, mangroves, sandy bays, seagrass beds, and soft-sediment habitats^40,42^. This shallow sea, connected to the Indian Ocean through the narrow Strait of Hormuz, is unique in its extreme environmental conditions. With an average depth of 35 m, salinity levels are high and often exceed 45 parts per thousand, while sea surface temperatures can fluctuate between 12 °C in winter to over 36 °C in summer^40^. The UAE is a coastal country located on the southern side of the Arabian Gulf with an Exclusive Economic Zone (EEZ) of 58,292 km^2^. It has a coastline of approximately 650 km facing the Arabian Gulf, and approximately 70 km bordering the Sea of Oman (Fig. 1).

### Sampling technique

The BRUVS dataset used here was not collected specifically to examine shark and ray diversity and abundance patterns. These surveys were conducted as a component of the Fisheries Resources Assessment Survey (FRAS) to assess the abundance and status of demersal fisheries resources in UAE Gulf waters. BRUVS were deployed from surface vessels during daytime hours at randomly generated stations (New Zealand National Institute of Water and Atmospheric Research (NIWA) Random Station Generator) across UAE Gulf waters. To avoid issues with maritime boundaries, surveys considered a 12 nautical mile (nm) exclusion zone inside of the UAE Exclusive Economic Zone and extended to 15 nm around the island of Abu Musa (Fig. 1a). Additionally, several restricted locations were excluded from the sampling (e.g., oilfields and concession areas, major shipping lanes and channels) as well as shallow lagoon areas or mangrove forests occurring at depths of 1–3 m that could not be accessed with FRAS vessels.

BRUVS were fitted with a GoPro Hero 4 + black high definition camera, set to the standard settings of high definition video quality (1080), super-wide frame and 25 frames per second) to ensure a full view of the seafloor, enclosed in a frame and mounted on a steel rod of 1.2 m height (Fig. 2). A weighed down (with 2 kg of dive weight on each side) 1 × 1 m piece of thick mesh (mesh dimension 2.5 × 2.5 cm) was used as a base frame for each BRUVS unit to stabilize it and ensure a consistent field of view. A bait arm made of two crossed 25 mm diameter PVC pipes was placed 1.5 m away from the center of the mesh. The bait consisted of 2 kg of fresh Indian oil sardines (*Sardinella longiceps*) in a wire mesh bag, chopped to maximize dispersal of the fish oil. BRUVS units were lowered to the sea bed from the boat using a rope with a buoy placed at the surface to facilitate manual retrieval. Cameras were set to record continuously for at least a 60 minute period.

### Video and data analysis

Species richness and relative abundance estimates of sharks and rays were recorded during the review of the video footage using Apple Quicktime by the first two authors. The start time of the video was recorded when BRUVS landed on the seafloor and settled in one location. To avoid repeat counts of individual sharks and rays continuously re-entering the field of view, the maximum number of individuals of the same species appearing at the same time (MaxN) was used as a relative abundance measure^43^. MaxN is a conservative estimate of abundance in high density areas^43,44^ and was recorded for five categories: Elasmobranchs (sharks and rays combined), Sharks, Rays, Arabian carpetshark (*Chiloscyllium arabicum*), and stingrays (*Himantura* spp.) which consisted of the reticulated whipray (*H*. *uarnak*) and the leopard whipray (*H*. *leoparda*). Deployment time, species, sex (if visible), time of first sighting, time of each subsequent sighting, MaxN, time of MaxN, and benthos type were recorded in all videos. Depth was determined using built-in vessel depth sounders at each site. Samples were discarded from analysis if horizontal visibility was low (<1.5 m), estimated by means of visibility of the bait bag.

Species were identified to the lowest possible taxon. If identification could not be confidently confirmed, then individuals were recorded under the closest genus or family name. For example, this occurred for individuals of the genus *Pastinachus* and *Rhinoptera*, where two look-alike species of each occur in Arabian Gulf waters and need close morphological examination in order to separate them. Furthermore, in some cases, sharks or rays made distant passes, making it difficult to identify to a family level. No attempts at measurements of individuals were made but coarse estimates of the total length (TL) or disc width (DW) of each individual were noted to the nearest 50 cm increment by comparing them with the length of the PVC pipe on the bait arm and individuals were classified as ‘adult’ or ‘juvenile’, based on their size compared to known maturity estimates from the region^24,25^.

All statistical analysis was conducted using SPSS (version 23). The catch per unit effort (CPUE), used as a relative abundance index, was calculated as Max N/hr^−1^ for each species and category. Relative abundance was compared for four factors: *Season* (Spring/Summer and Fall/Winter), *Depth* (shallow: 10 m or less and deep, over 10 m), *Geographic strata* (West, Central, and East), and *Habitat type* (Mud (unconsolidated sediment), Sand (consolidated sand), and Other (other habitats including seagrass, macro-algae, and coral assemblages)) using Kruskal-Wallis non-parametric rank tests (ANOVA) as CPUE data was found to deviate significantly from a normal distribution due to the abundance of 0 values within the data set. Significant differences among the groups were further explored using post-hoc Mann-Whitney U tests. A Bonferroni correction was not applied, given the radical lowering of statistical power and increased likelihood of Type II errors associated with this type of correction^12,45,46^.

The relationship between soak time and the number of species of elasmobranchs, sharks, and rays observed was evaluated by plotting a species cumulative curve against each 50-hour soak time interval. Finally, the CPUE was graphically compared to published estimates obtained using similar BRUVS approaches in terms of camera orientation (vertical to substrate), number of cameras (one at a time), bait type (pilchards or sardines), soak time (at least 60 mins), and video metrics used (MaxN), from different regions around the world^6,12,15,16,20,21,26,27,33^. Where more than one study has been conducted in the same region (i.e., Northwest Australia and Indonesia), the average of all published estimates was depicted^21^.

## Data Availability

The data that support the findings of this study are available from the Environment Agency–Abu Dhabi but restrictions apply to the availability of these data, which were used under license for the current study, and so are not publicly available. Data are however available from the authors upon reasonable request and with permission of the Environment Agency – Abu Dhabi.
